# Intestinal microbiota changes pre- and post-fecal microbiota transplantation for treatment of recurrent *Clostridioides difficile* infection among Iranian patients with concurrent inflammatory bowel disease

**DOI:** 10.3389/fmicb.2023.1147945

**Published:** 2023-02-24

**Authors:** Fahimeh Sadat Gholam-Mostafaei, Masoumeh Azimirad, Kaveh Naseri, Ali Nabavi-Rad, Hamid Asadzadeh Aghdaei, Shabnam Shahrokh, Nasser Ebrahimi Daryani, Abbas Yadegar, Mohammad Reza Zali

**Affiliations:** ^1^Basic and Molecular Epidemiology of Gastrointestinal Disorders Research Center, Research Institute for Gastroenterology and Liver Diseases, Shahid Beheshti University of Medical Sciences, Tehran, Iran; ^2^Foodborne and Waterborne Diseases Research Center, Research Institute for Gastroenterology and Liver Diseases, Shahid Beheshti University of Medical Sciences, Tehran, Iran; ^3^Gastroenterology and Liver Diseases Research Center, Research Institute for Gastroenterology and Liver Diseases, Shahid Beheshti University of Medical Sciences, Tehran, Iran; ^4^Department of Gastroenterology and Hepatology, Tehran University of Medical Sciences, Tehran, Iran

**Keywords:** *Clostridioides difficile* infection, fecal microbiota transplantation, recurrent CDI, inflammatory bowel disease, intestinal microbiota

## Abstract

**Introduction:**

Patients with inflammatory bowel disease (IBD) are at a greater risk for the recurrence of *Clostridioides difficile* infection (rCDI) that is triggered by intestinal microbiota dysbiosis. Fecal microbiota transplantation (FMT) has emerged as a highly effective therapeutic option for this complication. However, little is known about the impact of FMT on intestinal microbiota alterations in rCDI patients suffering from IBD. In this study, we aimed to investigate post-FMT intestinal microbiota alterations in Iranian rCDI patients with underlying IBD.

**Methods:**

A total of 21 fecal samples were collected including 14 samples pre- and post-FMT and 7 samples from healthy donors. Microbial analysis was performed by quantitative real-time PCR (RT-qPCR) assay targeting the 16S rRNA gene. The pre-FMT profile and composition of the fecal microbiota were compared to the microbial changes of samples collected 28 days after FMT.

**Results and discussion:**

Overall, the fecal microbiota profile of recipients was more similar to donor samples after the transplantation. We observed a significant increase in the relative abundance of Bacteroidetes post-FMT, compared to the pre-FMT microbial profile. Furthermore, there were remarkable differences between the microbial profile of pre-FMT, post-FMT, and healthy donor samples by PCoA analysis based on the ordination distance. This study demonstrates FMT as a safe and effective approach to restore the indigenous composition of the intestinal microbiota in rCDI patients and ultimately results in the treatment of concurrent IBD.

## Introduction

The human gastrointestinal tract harbors more than 10^14^ microorganisms including bacteria, archaea, fungi, and viruses that are collectively referred to as gut microbiota (Sender et al., 2016). It is now well established that healthy gut microbiota plays a crucial role in the host protection against enteric pathogens mainly through colonization resistance mechanism and can modulate both host innate and adaptive immune responses (Pickard et al., 2017; Rezasoltani et al., 2021). These notable features are critical in the healthcare settings to prevent hospital-acquired infections mainly derived from the gastrointestinal tract of hospitalized patients and the hospital environment (Kim et al., 2017; Ducarmon et al., 2019). However, certain environmental and genetic factors such as mode of birth, host genetics, age, diet, exposure to antibiotics, and impairment of the host immune system can influence the composition and function of the host gut microbiota and result in perturbations of microbiota structure (Hasan and Yang, 2019).

The destructive alterations in the diversity and composition of gut microbiota, known as gut dysbiosis, may predispose the host to severe intestinal tissue damage and inflammation-associated disorders, particularly inflammatory bowel disease (IBD), that increase the risk of certain pathological conditions like *Clostridioides difficile* infection (CDI) (Bien et al., 2013; Gholam-Mostafaei et al., 2020). During the last decade, there has been a significant rise in CDI incidence worldwide (Finn et al., 2021; Fu et al., 2021). Although antibiotics (especially vancomycin) are the mainstay to eradicate CDI, antibiotic supplementation may lead to perturbations of the patient’s gut microbiota and can perpetuate the recurrence of CDI (rCDI) (Sehgal and Khanna, 2021). Globally, about 10–20% (with a median recurrence rate of 17%) of incident CDI patients experience recurrence after an initial course of anti-CDI therapy, and the risk of relapse increases with each episode of recurrence (Finn et al., 2021; Kelly et al., 2021). Furthermore, it is estimated that the prevalence of CDI in the IBD population is 2.5 to 8-fold higher than those without IBD, which may worsen the disease course, and subsequently lead to an IBD flare (Kelsen et al., 2011; Hourigan et al., 2014).

Over the past decade, research on fecal microbiota transplantation (FMT) has blossomed, and its safety and efficacy in treating rCDI have been approved by the American College of Gastroenterology in the latest guidelines (Kelly et al., 2021). There is so much evidence supporting that FMT restores the normal composition and functionality of the fecal microbial community to the phylogenetic diversity levels more typical of a healthy person and has emerged as a highly effective therapeutic approach that successfully treats >90% of patients suffering from rCDI with negligible adverse events (AEs) (Weingarden et al., 2014; Fischer et al., 2016). There is also emerging evidence that FMT may be safe and efficacious in treating rCDI and potentially leads to improvement of the underlying pathophysiology in patients with concurrent IBD (Fischer et al., 2016; Chin et al., 2017; Allegretti et al., 2020). However, the definite mechanisms and likely scenarios by which this transplanted microbiota exerts its therapeutic effects and prevents the recurrence of CDI, particularly in patients with IBD flare, have not yet been fully elucidated.

Recently, in our prior research, we documented that FMT could be an efficient and safe therapeutic option for the resolution of rCDI in Iranian patients with underlying IBD (Gholam-Mostafaei et al., 2021). However, little information is available about the impact of FMT on gut microbiota alterations in rCDI patients with IBD. Here we aimed to assess how intestinal microbiota changes in Iranian rCDI patients with underlying IBD before and after FMT.

## Materials and methods

### Patients, donors, and fecal specimens

**FIGURE 1:**
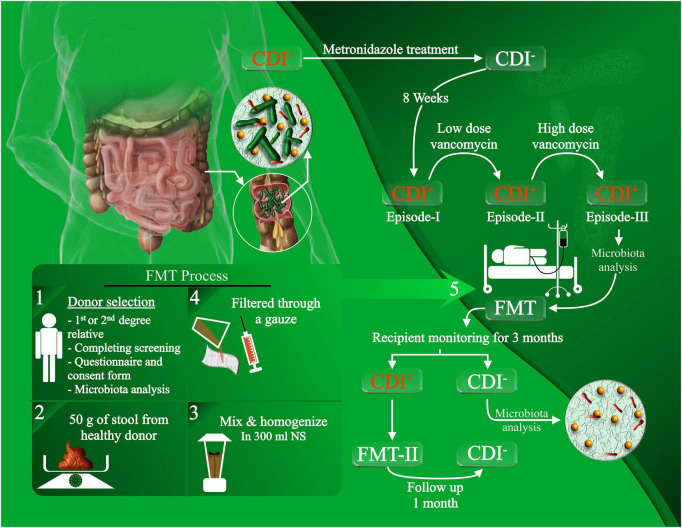
Experimental design of the FMT procedure and patient recruitment. rCDI patients with concurrent IBD, who failed to eradicate the infection with antibiotic treatments, were recruited for FMT administration. The FMT procedure was conducted by selecting healthy donors, filtering and homogenizing the fecal material, and infusing the fecal suspension through colonoscopy. Stool samples were collected from the donors and recipients for microbiota analysis prior to FMT and after the follow-up.

All patients recruited into this study suffered from rCDI with underlying IBD and failed to eradicate the infection, despite several rounds of antibiotic therapies as presented in our previous work (Gholam-Mostafaei et al., 2021). Healthy stool donors were rigorously screened and included genetically related, patient-oriented first-degree and third-degree relatives, who donated freshly passed fecal materials on the day of transplantation, which was rapidly processed within 6 h of defecation. None of the donor fecal specimens were frozen or banked. Stool samples from the recipients were collected prior to FMT (pre-FMT) and at day 28 (post-FMT), and were stored in aliquots at –80°C until further analysis (Figure 1). FMT procedure was carried out via colonoscopy, and the fecal suspension was infused into the terminal ileum or cecum of the patients as previously described (Gholam-Mostafaei et al., 2021).

### Consent to participation

All enrolled patients provided written informed consent prior to the FMT procedure and their clinical data and stool samples were collected. Eligible patients were adults (≥18 years) and required to have documentation of typical histopathology for IBD with colonic involvement and a confirmed diagnosis of at least three or more documented rCDI with typical laboratory findings. The study protocol conformed to the ethical guidelines of the Institutional Ethical Review Committee of the Research Institute for Gastroenterology and Liver Diseases at Shahid Beheshti University of Medical Sciences (Project No. IR.SBMU.RIGLD.REC.1398.036).

### DNA preparation

According to the manufacturer’s instructions with some modifications, total DNA was extracted from the stool samples of donors and recipients using the QIAamp DNA Stool Mini Kit (Qiagen Retsch GmbH, Hannover, Germany). The concentration and quality of purified DNA samples were assessed by NanoDrop ND-2000 Spectrophotometer (NanoDrop products, Wilmington, DE, USA) and electrophoresis on 0.8% (w/v) agarose gels. Extracted DNA samples were stored at –20°C until used for further molecular analysis.

### Gut microbiota profiling by quantitative real-time PCR

Oligonucleotide primers used for determining universal and group-specific bacterial taxa based on the bacterial 16S rRNA sequences are listed in Table 1. The different microbiota phyla/classes/genera examined in this study were selected based on the literature review and Disbiome database^1^ for microbial composition alterations in the feces of patients with IBD compared with related healthy donors (Janssens et al., 2018). Briefly, each PCR reaction was performed in a final volume of 20 μL, comprising 10 μL of SYBR green PCR master mix (Ampliqon, Odense, Denmark), 1 μL of 10 pmol of forward and reverse primers, and 100 ng of the DNA template. The temperature profile for each bacterial taxonomic group’s amplification was indicated in our previous works (Naseri et al., 2021; Ostadmohammadi et al., 2021). All PCR amplifications were performed in triplicate using a Rotor-Gene^®^ Q (Qiagen, Germany) real-time PCR system. The amplification specificity was determined by the melting curve analysis with increasing temperature from 60 to 95°C (at a regular increment of 0.5°C for 5 s). The relative abundance of each bacterial taxonomic group among patients pre-FMT, post-FMT, and the corresponding healthy donors was calculated according to the method described previously by Bacchetti De Gregoris et al. (2011). Accordingly, the average Ct value obtained from each primer pair was transformed into a percentage using the following formula:


$$X=\frac{{\left(\mathrm{Eff}.\mathrm{Univ}\right)}^{\text{Ct}\,\text{univ}}}{{\left(\mathrm{Eff}.\mathrm{Spec}\right)}^{\text{Ct}\,\text{spec}}}\times 100$$

**TABLE 1:** The gut microbiota taxon-specific primers used in this study.

Target taxon	Primer name	Primer sequence (5′–3′)	Amplicon length (bp)	References
*Eubacteria*	UniF340 UniR514	ACTCCTACGGGAGGCAGCAGT ATTACCGCGGCTGCTGGC	∼ 200	de Moraes et al., 2016
Firmicutes	Firm934-F Firm1060-R	GGAG**Y**ATGTGGTTTAATTCGAAGCA AGCTGACGACAACCATGCAC	∼ 129	Matsuki et al., 2004
Bacteroidetes	Bac960-F Bac1100-R	GTTTAATTCGATGATACGCG TTAAGCCGACACCTCACG	∼ 137	Matsuki et al., 2004
Actinobacteria	Actino-F Actino-R	GCG**K**CCTATCAGCTTGTTGGTG CCGCCTACGAGC**Y**CTTTACGC	∼ 333	Hermann-Bank et al., 2013
Fusobacteria	Fuso-F Fuso-R	GATCCAGCAATTCTGTGTG CGAATTTCACCTCTACACTTG	∼ 290	Keshavarz Azizi Raftar et al., 2021
Tenericutes	Ten662-F Ten862-R	ATGTGTAGCGGTAAAATGCGTAA C**M**TACTTGCGTACGTACTACT	∼ 219	Yang et al., 2015
Verrucomicrobia	Ver1165-F Verru-R	TCA**K**GTCAGTATGGCCCTTA GCAGCCTACAGTCCGAAC	∼ 123	Yang et al., 2015
Spirochaetes	Spiro-F Spiro-R	GT**Y**TTAAGCATGCAAGTC AATATTCTTAGCTGCTGCC	∼ 321	This study
Alpha-proteobacteria	α682-F α968-R	CGAGTGTAGAGGTGAAATTC GGTAAGGTTCTGCGCGTT	∼ 305	Keshavarz Azizi Raftar et al., 2021
Beta-proteobacteria	Beta979-F Beta1130-R	AACGCGAAAAACCTTACCTAC GCCCTTTCGTAGCAACTA	∼ 175	Yang et al., 2015
Gamma-proteobacteria	Gamma395-F Gamma871-R	C**M**ATGCCGCGTGTGTGAA ACTCCCCAGGCGGTC**D**ACTTA	∼ 498	Bacchetti De Gregoris et al., 2011
Delta-proteobacteria	Gamma877-F Gamma1066-R	GCTAACGCATTAAGT**RY**CCCG TGACGACAGCCATGCAGCACC	∼ 210	Yang et al., 2015
Epsilon-proteobacteria	Epsilon-F Epsilon-R	TGGTGTAGGGGTAAAATCCG AGGTAAGGTTCTTCG**Y**GTATC	∼ 286	Hermann-Bank et al., 2013
*Clostridium* cluster I	Chis150-F ClostI-R	AAAGGAAGATTAATACCGCATA TTCTTCCTAATCTCTACGCA	∼538	Ostadmohammadi et al., 2021
Enterobacteriaceae	Enterob-F Enterob-R	CGTCGCAAG**MM**CAAAGAG TTACCGCGGCTGCTGGCAC	∼ 351	Hermann-Bank et al., 2013
*Clostridium coccoides* group	g-Ccoc-F g-Ccoc-R	AAATGACGGTACCTGACTA CTTTGAGTTTCATTCTTGCGA	∼ 438	Matsuki et al., 2002
*Streptococcus* spp.	Str1-F Str2-R	GTACAGTTGCTTCAGGACGT GTTCGATTTC**R**TCACGTTG	∼ 195	Hermann-Bank et al., 2013
*Ruminococcus* spp.	Rflbr730F Clep866mR§	GGCGGC**Y**T**R**CTGGGCTTT GCAGGTGGAT**W**ACTTATTGTGTTAA	∼ 157	Hermann-Bank et al., 2013
*Prevotella* spp.	g-Prevo-F g-Prevo-R	CAC**R**GTAAACGATGGATGC TTGCAGACCCCAGTCCGAAC	∼ 507	This study
*Bifidobacterium* spp.	Bifid-F Bifid-R	GGGATGCTGGTGTGGAAGAG TGCTCGCGTCCACTATCCAG	∼ 200	Wang et al., 2012
*Lactobacillus* spp.	Lacto-F Lacto-R	TGGATGCCTTGGCACTAG AAATCTCCGGATCAAAGCTTAC	∼ 89	Wang et al., 2012
*Enterococcus* spp.	Str1-F Str2-R	GTACAGTTGCTTCAGGACGT GTTCGATTTC**R**TCACGTTG	∼ 195	Wang et al., 2012
*Staphylococcus* spp.	Staph-F Staph-R	GAACGTGGTCAAATCAAAG CAACACCAGTTACGTCAGTAG	∼ 328	Martineau et al., 2001
*Salmonella* spp.	Sal-F Sal-R	CATTTCTATGTTCGTCATTCCATTACC AGGAAACGTTGAAAAACTGAGGATTCT	∼ 132	This study
*Klebsiella* spp.	Kleb-F Kleb-R	TCCTGGCTCAGATTGAACGC AGACATTACTCACCCGTCCG	∼ 107	This study

The nucleotides in bold type represent Y, C or T; K, G or T; M, A or C; D, A or G or T; R, A or G.

where the Eff. Univ refers to the calculated efficiency of the universal primers for *Eubacteria* (2 = 100% and 1 = 0%) and Eff. Spec indicates the efficiency of the taxon-specific primers. Ct univ and Ct spec represent the threshold cycles registered by the thermocycler. “X” addresses the percentage (%) of taxon-specific 16S rRNA gene copy numbers in an individual fecal sample.

### Statistical analysis

The descriptive statistics were provided for quantitative and categorical variables as mean and standard deviation (SD) or frequencies (n), respectively. After testing for normality by applying the Shapiro–Wilk test, independent sample t-test and paired sample t-test were used for the parametric data analysis, and Mann–Whitney and Wilcoxon signed-rank tests were used for non-parametric data analysis. Statistical Package for Social Sciences software (version 24, SPSS Inc., Chicago, USA) was applied for running statistical analysis. The principal component analysis (PCA) was conducted to determine the patterns of variability in the data using the FactoMineR package from the open-source statistical program R version 3.6.1 (R Core Team, Vienna, Austria). The principal coordinate analysis (PCoA) was calculated using the same program based on the Bray-Curtis dissimilarity method (Bray and Curtis, 1957). Spearman’s correlation was used for non-parametric analysis. The graphs were prepared using GraphPad Prism software version 5.04 (GraphPad Software, San Diego, CA, USA) and the open-source statistical program R version 3.6.1 (R Core Team, Vienna, Austria). A *P* value less than 0.05 was considered statistically significant.

## Results

### Patients, donors, and clinical outcomes

Seven IBD patients with rCDI and their related donors were enrolled in this study. All IBD patients had a history of 3 episodes of CDI and failed the standard antibiotic therapies. The median patients’ age was 31.5 years including three females and four males, and the median BMI was 24.5 in pre-FMT. According to our FMT protocol, the donors were screened as previously described (Gholam-Mostafaei et al., 2021). The clinical outcomes in patients with rCDI were defined as negative PCR results and CDI symptom resolution and CDI recurrence. Symptoms of CDI, including diarrhea, abdominal pain, and bowel movements resolved in all patients within two months follow-up of post-FMT. Furthermore, the PCR results demonstrated complete resolution of CDI in all 7 patients. On patient, however, presented late *C. difficile* recurrence after 18 months of follow-up, which was enrolled for second FMT and a successful treatment of rCDI was eventually achieved. The detailed characteristics and medication of the patients involved in this study were reported in our previous investigation (Gholam-Mostafaei et al., 2021).

Totally, 21 fecal samples consisting of 7 samples from FMT donors, 7 samples from rCDI patients with IBD pre-FMT, and 7 samples from the same patients post-FMT were collected for intestinal microbiota composition analysis. Post-FMT samples were collected 28 days after the procedure and the relative abundance for each bacterial taxon was calculated to conduct appropriate analyses and obtain *P* values as shown in Table 2.

**TABLE 2:** Relative abundance of the bacterial taxa (at the phylum, class, and genus level) srudied in this work.

Microbiota taxa	Pre-FMT	Post-FMT	*P*-value*	Donors
**Phylum**				
Firmicutes	24.13 ± 11.30	17.20 ± 6.25	0.043*	12.82 ± 6.01
Bacteroidetes	11.89 ± 11.68	19.36 ± 11.63	0.018*	25.79 ± 9.43
Actinobacteria	7.51 ± 6.49	7.67 ± 3.89	0.735	8.04 ± 5.50
Fusobacteria	3.10 ± 2.52	2.86 ± 1.26	0.735	4.52 ± 3.612
Tenericutes	1.27 ± 1.58	0.72 ± 0.79	0.735	1.32 ± 1.47
Verrucomicrobia	0.69 ± 0.85	0.27 ± 0.32	0.310	1.64 ± 0.98
Spirochaetes	0.46 ± 0.47	0.08 ± 0.07	0.063**	0.10 ± 0.08
**Class**				
Alpha-Proteobacteria	1.19 ± 1.44	2.15 ± 1.23	0.018*	1.88 ± 0.94
Beta-Proteobacteria	0.30 ± 0.50	0.99 ± 0.69	0.018*	2.97 ± 1.35
Gamma-Proteobacteria	1.21 ± 1.07	1.19 ± 0.74	0.612	0.77 ± 0.73
Delta-Proteobacteria	1.83 ± 0.92	1.24 ± 0.67	0.028*	0.70 ± 0.33
Epsilon-Proteobacteria	1.39 ± 0.69	1.20 ± 0.58	0.612	0.77 ± 0.41
Clostridia cluster I	1.31 ± 0.68	1.08 ± 0.70	0.310	0.92 ± 0.55
**Genus**				
*Enterococcus* spp.	2.62 ± 2.72	1.66 ± 1.59	0.499	1.10 ± 0.98
*Streptococcus* spp.	2.13 ± 1.60	0.92 ± 0.91	0.128	1.16 ± 0.88
*Bifidobacterium* spp.	1.50 ± 1.54	1.32 ± 0.57	0.310	1.66 ± 1.03
*Clostridium coccoides* group	1.50 ± 0.83	0.81 ± 0.48	0.028**	0.56 ± 0.55
*Ruminococcus* spp.	0.88 ± 0.51	0.85 ± 0.56	1.00	0.44 ± 0.64
*Lactobacillus* spp.	0.65 ± 0.52	0.94 ± 0.51	0.091**	0.84 ± 0.41
*Prevotella* spp.	0.48 ± 0.46	0.60 ± 0.56	0.237	0.63 ± 0.58
*Salmonella* spp.	0.28 ± 0.25	0.42 ± 0.39	0.398	0.13 ± 0.15
*Klebsiella* spp.	0.19 ± 0.30	0.01 ± 0.02	0.499	0.02 ± 0.05
*Staphylococcus* spp.	0.02 ± 0.04	0.05 ± 0.08	0.499	0.23 ± 0.24

Values are means ± SDs. Obtained from Wilcoxon signed-rank analysis. Differences were considered statistically significant when *P* < 0.05; *P < 0.05, and **P < 0.01.

### Composition of fecal communities in donors and patients before FMT procedure

**FIGURE 2:**
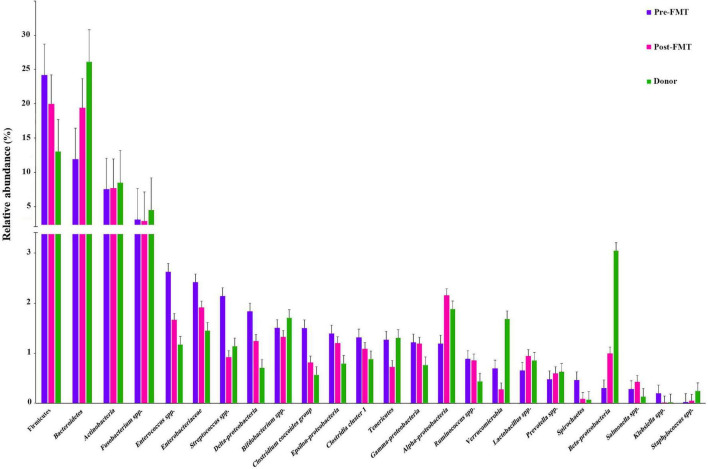
Bar-plot analysis of the relative abundance and distribution of each targeted gut microbiota in fecal samples of healthy donors, and patients before (pre-FMT) and after (post-FMT) fecal microbiota transplantation (FMT). Data are presented as mean ± SD.

**FIGURE 3:**
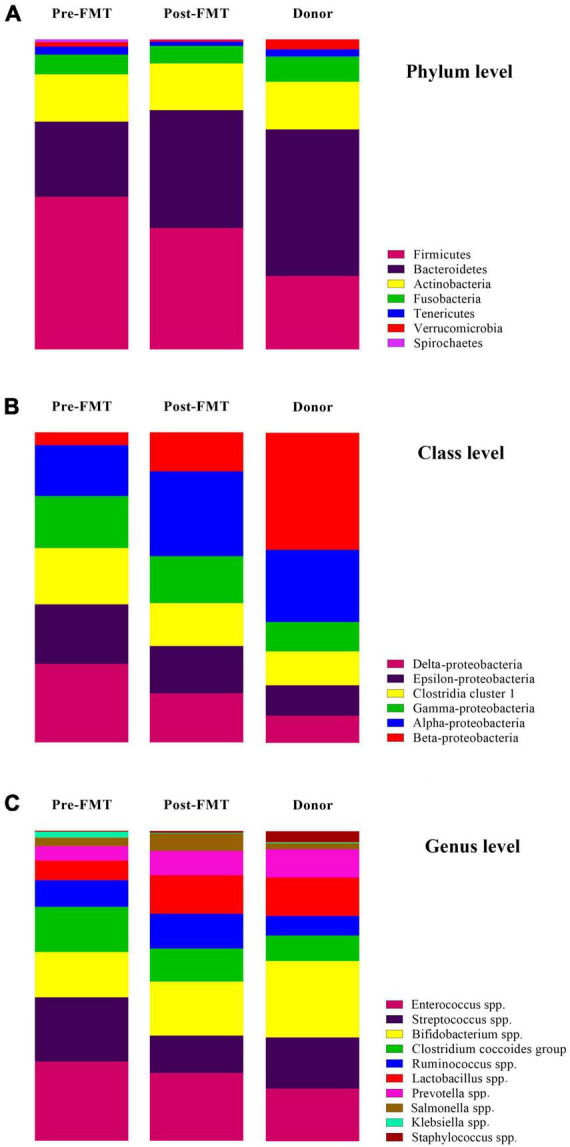
The relative percentage and changes of targeted gut microbiota communities in fecal samples of healthy donors, and patients before (pre-FMT) and after (post-FMT) fecal microbiota transplantation (FMT). **(A)** Gut microbiota composition and alterations at the phylum level. **(B)** Gut microbiota composition and alterations at the class level. **(C)** Gut microbiota composition and alterations at the genus level. Data are presented as mean ± SD. Each color corresponds to a type of microbiota included in this study.

Many differences were observed at the phylum, class, and genus levels among pre-FMT, post-FMT, and donor samples (Figure 2 and Supplementary Figure 1). The most abundant phyla in pre-FMT samples were Firmicutes, while the phylum Bacteroidetes had the highest abundance in post-FMT and donors. Delta-Proteobacteria was the most abundant one at the class level in the pre-FMT samples. In contrast, the class Beta-Proteobacteria had the lowest percentage among all the studied classes in the pre-FMT samples, while it contributed to the highest percentage in the donors. Furthermore, the donor samples had a lower relative abundance of Epsilon-Proteobacteria and Delta-Proteobacteria than the pre-FMT. In comparing donors and patients in pre-FMT, our results show that both *Bifidobacterium* spp. and *Lactobacillus* spp. were more abundant in donors, and the frequency of *Enterococcus* spp. and *Streptococcus* spp. was higher in pre-FMT patients (Figure 3).

### Composition of fecal communities in patients following FMT procedure

**FIGURE 4:**
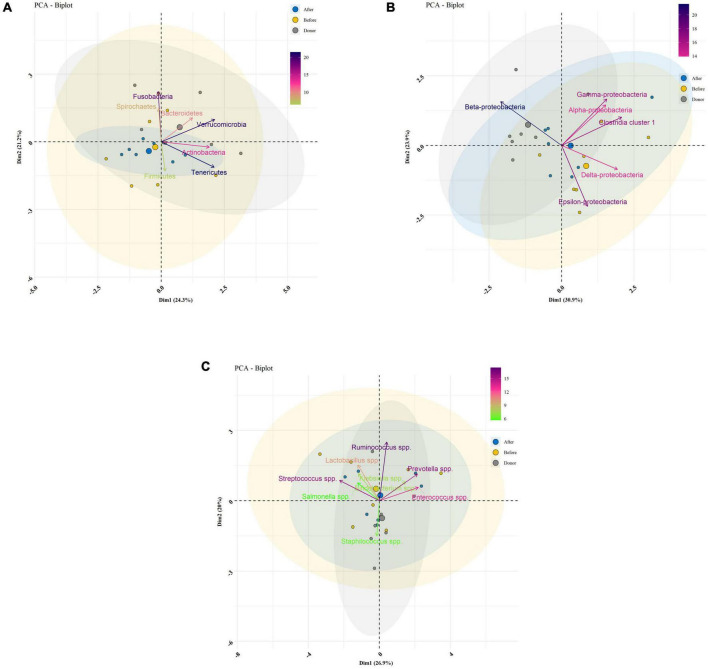
Bacterial community clustering and variations using principal component analysis (PCA) in fecal samples of healthy donors, and patients before (pre-FMT) and after (post-FMT) fecal microbiota transplantation (FMT). **(A)** PCA of gut microbiota clustering at the phylum level. **(B)** PCA of gut microbiota clustering at the class level. **(C)** PCA of gut microbiota clustering at the genus level. Percentage values in parentheses next to Dim1 and Dim2 represent the percentage of variance explained by each component. Arrows show the contribution of each type of microbiota on Dim1 and Dim2. Each data point denotes an individual patient, colored based on their group.

Descriptive statistics for the predominant bacteria showed that microbial community composition in post-FMT was shifted toward that of the donor at baseline, and the microbial community structure among post-FMT samples significantly changed in comparison to pre-FMT on 28 days after transplantation (Table 2). Bacteroidetes were the most abundant phylum in donor samples, and their abundance was significantly increased in post-FMT (*P* = 0.018). In addition, PCA analysis based on the ordination distance revealed an overall difference in the microbial compositions between pre-FMT, post-FMT, and donor samples as schematically depicted in Figure 4.

### Firmicutes to Bacteroidetes ratio

**FIGURE 5:**
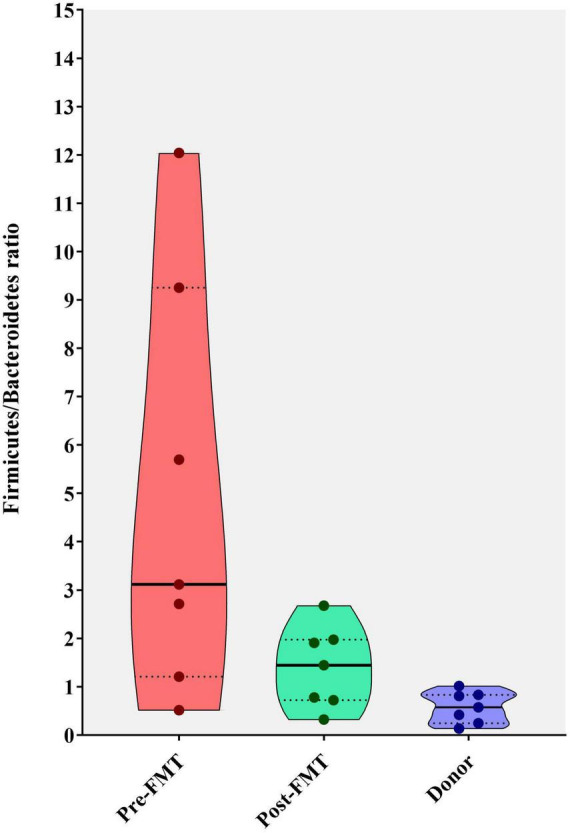
Violin plots showing the Firmicutes to Bacteroidetes (F/B) ratio in fecal samples of healthy donors, and patients before (pre-FMT) and after (post-FMT) fecal microbiota transplantation (FMT). This ratio was significantly (*P* = 0.001) decreased following the FMT (post-FMT) compared with pre-FMT using Mann–Whitney test.

The calculation of the Firmicutes/Bacteroidetes ratio is widely used to determine the normal homeostasis in the gut microbiota composition analysis. In the current study, we used it to show the degree of dysbiosis in the pre-FMT and post-FMT groups compared with the donors. This ratio significantly reduced in the post-FMT group, getting closer to the donors’ microbial composition during 28 days as depicted in Figure 5.

### Dissimilarity and principal coordinates analysis

**FIGURE 6:**
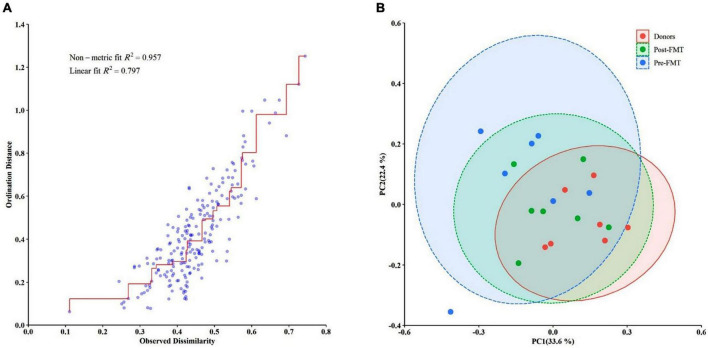
Differences in the gut microbiota composition of the studied groups using the Bray–Curtis dissimilarity metric plotted in a principal coordinate analysis (PCoA) space. **(A)** The Bray–Curtis dissimilarity plot. **(B)** The PCoA comparing the microbial communities from different groups (pre-FMT, post-FMT, and donors). Each circle represents a participant colored according to the studied group. Spearman’s correlation was used for non-parametric analysis.

We measured the extent of fit of the ordination by plotting the observed dissimilarity (as calculated by the dissimilarity matrix) to the ordination distance using a shepherd plot, which yielded a linear fit *R*^2^ = 0.797, indicating a good fit between the ordination distance and the observed dissimilarity, as calculated by Bray–Curtis index (Figure 6A). Moreover, the dissimilarity between the microbiota changes of different groups is shown in Figure 6B. PCoA suggested that post-FMT samples shifted more similarly to donors in the context of gut microbiota representing the effectiveness of the FMT procedure in the present study.

## Discussion

Given the dysbiotic characteristics of the intestinal microbiota in IBD patients, these individuals are at higher risk of infection with *C. difficile* and developing rCDI (Azimirad et al., 2020, 2022). The standard antibiotic treatment for initial infection with *C. difficile* and rCDI among IBD patients includes metronidazole and vancomycin therapy. Over the last few years, FMT has appeared as a highly effective treatment for CDI and especially rCDI patients, which are refractory to conventional antibiotic therapy. In this study, we investigated alterations in the intestinal microbiota composition of Iranian rCDI patients with underlying IBD 28 days following FMT intervention. Overall, our results demonstrated a structural modification of the intestinal microbial community, resembling that of the healthy donors. These findings are in agreement with a previous study, in which FMT led to a substantial alteration in microbiota diversity of rCDI patients along with the complete resolution of IBD symptoms (Khanna et al., 2017). However, several studies have shown that FMT is more effective in CDI patients without IBD than those with concurrent IBD (Khoruts et al., 2016; Chin et al., 2017). In our previous study, the effectiveness of the initial FMT for rCDI treatment in IBD patients was 100%, which presented a complete resolution of diarrhea and CDI-negative status, 2 months after a single FMT (Gholam-Mostafaei et al., 2021).

In the present study, post-FMT fecal microbiota analysis at the phylum level demonstrated significant differences in the composition of Firmicutes, Bacteroidetes, and Spirochaetes phyla, compared to the pre-FMT microbial profile. Compared to healthy donors and post-FMT microbiota, IBD patients had a significantly higher Firmicutes/Bacteroidetes ratio, which could accelerate rCDI development as some reports show an association between CDAD and increased Firmicutes/Bacteroidetes ratio in the intestinal microbiota composition (Manges et al., 2010; Ling et al., 2014). Our results indicated that the relative abundance of phylum Firmicutes significantly decreased post-FMT, similar to that of the donor microbiota. However, this finding is in contrast with the results of previous studies in which the abundance of phylum Firmicutes increased after bacteriotherapy or FMT in *C. difficile*-associated disease (CDAD) (Khoruts et al., 2010), and rCDI (Weingarden et al., 2014), respectively. Furthermore, microbiota analysis results exhibited a great abundance of Firmicutes phyla in pre-FMT samples, which is in contrast to previous reports indicating the higher abundance of Proteobacteria and Bacteroidetes in the intestinal microbiota of rCDI patients with (Khanna et al., 2017) and without IBD (Chang et al., 2008; Khoruts et al., 2010), respectively. Also, Firmicutes and Bacteroidetes are the two predominant phyla among the gut microbiota (Micah et al., 2007) and several studies have reported imbalances for both phyla in IBD patients (Zhou and Zhi, 2016; Bernstein and Forbes, 2017; Alam et al., 2020). In a similar study conducted by Khanna et al., substantial alterations were observed in the intestinal microbial composition of rCDI patients toward the donor microbiota profile along with an overall increase in microbial diversity following the FMT procedure (Khanna et al., 2017). They also reported a significant increase in the relative abundance of *Bacteroides* and *Faecalibacterium* genera in both rCDI groups with or without concurrent IBD; however, only a smaller group of microbiota taxa significantly altered 7 days after FMT in rCDI patients with concurrent IBD. Similarly, Khoruts et al. reported that the fecal microbial composition of the patients with CDAD was greatly shifted toward that of the healthy donor and was dominated by *Bacteroides* spp. strains and an uncharacterized butyrate-producing bacterium (TRF 274), 14 days post-transplantation (Khoruts et al., 2010).

At the class level in the pre-FMT microbial composition, the delta-Proteobacteria was the most abundant bacteria, which are largely found in UC patients. These bacteria can cause colonic mucosal injury in UC patients by producing hydrogen sulfide (Mukhopadhya et al., 2012). Our results further demonstrated that both the *Bifidobacterium* spp. and *Lactobacillus* spp. were more abundant in donor samples, compared to rCDI patients. Given the significant reduction of beneficial *Lactobacillus* species in both UC and CD patients, an increased abundance of these bacteria after the FMT procedure might contribute to a favorable response to FMT and subsequent induction of clinical remission. These bacteria are responsible for carbohydrate fermentation in the colon and the production of short-chain fatty acids (SCFAs) including butyrate, propionate, and acetate (Mańkowska-Wierzbicka et al., 2020). Some strains of Lactobacilli may lower the abundance of *Bifidobacterium* and the production of pro-inflammatory cytokines, thereby facilitating the remission of clinical symptoms (Cristofori et al., 2021).

This study aimed to compare the fecal microbiota composition before and after FMT in IBD patients with rCDI and evaluated FMT efficacy for rCDI treatment. The small sample size was the main constraint for a more powerful analysis in the current study. Also, several FMT procedures have been developed to increase FMT efficacy, while in our study, only fresh stools were examined using a colonoscopy. Furthermore, this study is limited to RT-qPCR data analysis, and high throughput sequencing techniques such as metagenomics are neglected.

## Conclusion

This study demonstrated a significant alteration in the microbial composition of IBD patients with rCDI after FMT. Our results, in principle, represented FMT as an effective and safe therapeutic strategy to restore the inherent microbial composition in IBD patients. Considering microbial dysbiosis in IBD patients and the potential for intestinal permeability and microbial co-infection, restoring intestinal homeostasis and integrity appears mandatory in the course of IBD. Therefore, FMT might be a fruitful approach for the clinical management of rCDI and concurrent IBD. However, large population cohort studies with multi-omics approaches are required to evaluate and analyze metagenomic, metatranscriptomic, and metabolomic profiles of the intestinal microbiota in IBD patients post-FMT.

## Data availability statement

The original contributions presented in this study are included in the article/Supplementary material, further inquiries can be directed to the corresponding author.

## Ethics statement

The studies involving human participants were reviewed and approved by Institutional Ethical Review Committee of Research Institute for Gastroenterology and Liver Diseases at Shahid Beheshti University of Medical Sciences (Project No. IR.SBMU.RIGLD.REC.1398.036). The patients/participants provided their written informed consent to participate in this study.

## Author contributions

FG-M and MA performed the microbiological screening tests, prepared the stool suspension, and carried out microbiota analysis experiments. AY designed the study, reviewed the literature, and supervised the project. AY and KN conducted the microbiota and statistical analyses. AY and AN-R wrote the manuscript. HA, SS, and NE performed the colonoscopic procedures. AY and MZ critically revised the manuscript. All authors contributed to the article and approved the submitted version.

## Footnotes

1
https://disbiome.ugent.be/home
