# Supplementary material for: Intestinal microbiota changes pre- and post-fecal microbiota transplantation for treatment of recurrent Clostridioides difficile infection among Iranian patients with concurrent inflammatory bowel disease
Source: Front Microbiol. 2023 Feb 24;14:1147945. doi: 10.3389/fmicb.2023.1147945 (PMC9998922; doi:10.3389/fmicb.2023.1147945)
Supplement: Supplementary file 1 [file Data_Sheet_1.docx]

**
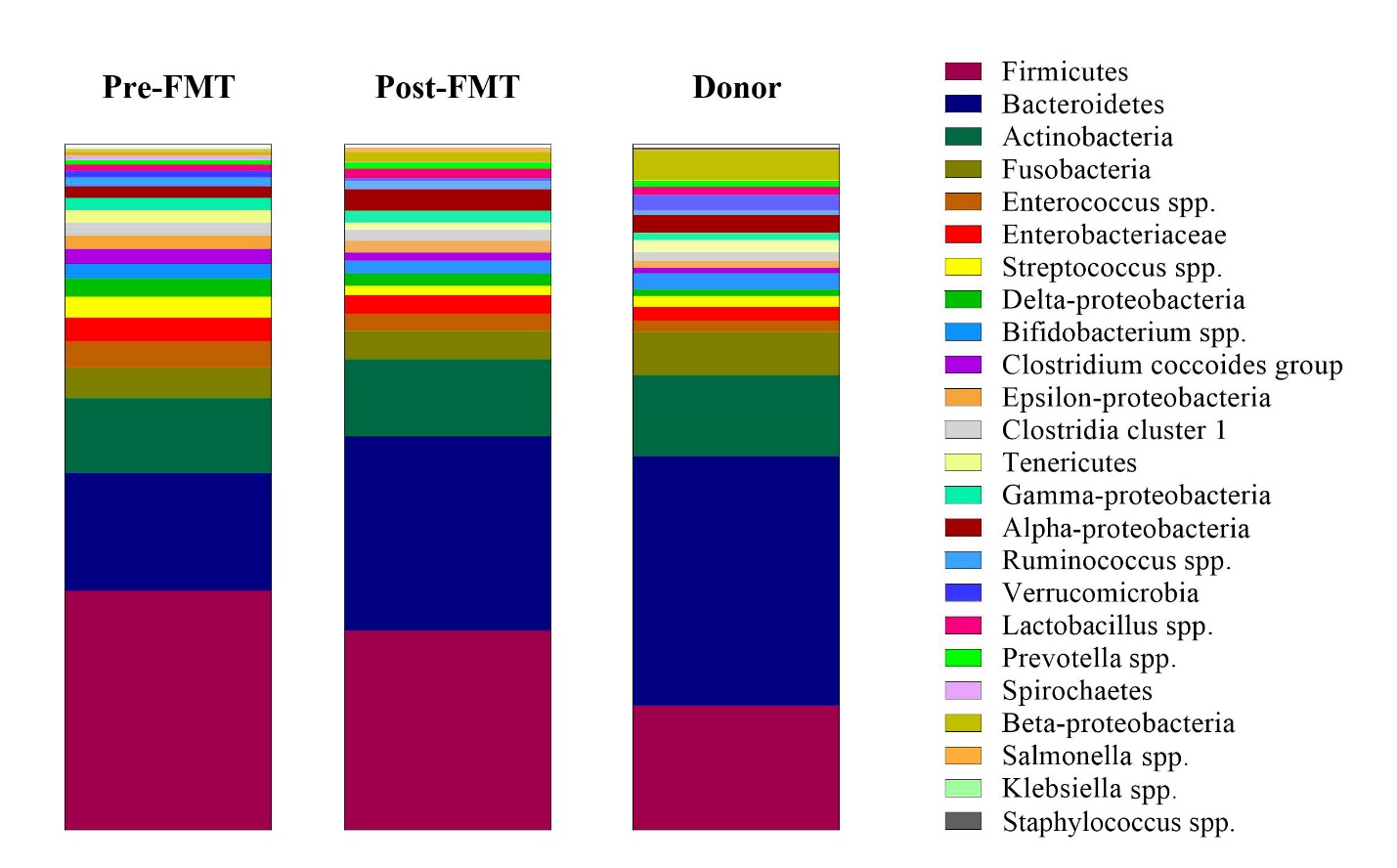
**

**Figure S1.** The relative percentage and changes of targeted gut microbiota communities in fecal samples of healthy donors, and patients before (pre-FMT) and after (post-FMT) fecal microbiota transplantation (FMT). Data are presented as mean ± SD. Each color corresponds to a type of microbiota included in this study.
